# Supplementary material for: Bumblebees moving up: shifts in elevation ranges in the Pyrenees over 115 years
Source: Proc Biol Sci. 2020 Nov 11;287(1938):20202201. doi: 10.1098/rspb.2020.2201 (PMC7735265; doi:10.1098/rspb.2020.2201)
Supplement: Appendix: Sampling Protocol 1889 [file rspb20202201supp1.docx]

**Supplementary Material – Appendix A**

Article DOI: **10.1098/rspb.2020.2201**

Article: **Bumblebees moving up: shifts in elevation ranges in the Pyrenees over 115 years**

Journal: **Proceedings of the Royal Society B: Biological Sciences**

Author list: Leon Marshall*, Floor Perdijk, Nicolas Dendoncker, William Kunin, Stuart Roberts, Koos Biesmeijer

* Corresponding author

**List of sites sampled in 1889 from “De Pyreneeënbloemen en hare bevruchting door insecten” by J. MacLeod published in 1891, interpreted from Dutch into English by Koos Biesmeijer.**

MacLeod spent August 5 to 31 in 1889 in the area around Gèdre in the Hautes Pyrenees, France to assess which insects were visiting the flowering plants. Most time was spent between 1000m (around Gèdre) and 1500m (cascade de Gavarnie), with some visits to higher areas (Gavarnie up to 2300m, Cirque du Troumouse (up to 2000m) and one visit to the Brêche de Roland (2800m).

The short description of the area can be summarized as follows: The Luz valley around Gèdre consisted pastures and arable fields, with some low shrubs on the hillsides, but no forest. The whole area from 900-1300 was very rich in flowers in August particularly on the gravel beds along the river and on the extensive rock beds (‘chaos’) in the area. The meadows were mostly mown off in August (normal practice being a first cut in June, second on in July and sometimes third cut in August).

Around the Gavarnie area they found flora with alpine character. At the base of the Cirque du Gavarnie (1600-1700m), they still found snow in August with abundant alpine flora. There were some pine and fir forests present between the alpine meadows. The presence of alpine flora at such a low elevation is a result of the high walls of Cirque du Gavarnie (400-500m high) on the south side, preventing warm southern winds and limiting sunlight reaching the soil.

The Cirque du Troumouse was visited from Gèdre following the road to Héas (around 1500m) and on to Troumouse (up to 2000m). The area was heavily grazed (‘thousands of sheep’) and few flowers were left in Troumouse. Only poisonous Aconitums and spiny thistles (e.g. Carduus carlinoides) were flowering abundantly. Despite the overgrazing, they observed abundant insect life of mostly small species.

The plateau of Saugué (1500-1650m) consisted of hay meadows (‘the best in the region’).
