# Supplementary material for: Bumblebees moving up: shifts in elevation ranges in the Pyrenees over 115 years
Source: Proc Biol Sci. 2020 Nov 11;287(1938):20202201. doi: 10.1098/rspb.2020.2201 (PMC7735265; doi:10.1098/rspb.2020.2201)
Supplement: Supplementary Figure: Climate and land use change results, and species accumulation curves [file rspb20202201supp2.docx]

**Supplementary Figures**

Article DOI: **10.1098/rspb.2020.2201**

Article: **Bumblebees moving up: shifts in elevation ranges in the Pyrenees over 115 years**

Journal: **Proceedings of the Royal Society B: Biological Sciences**

Author list: Leon Marshall*, Floor Perdijk, Nicolas Dendoncker, William Kunin, Stuart Roberts, Koos Biesmeijer

* Corresponding author

**Figures**


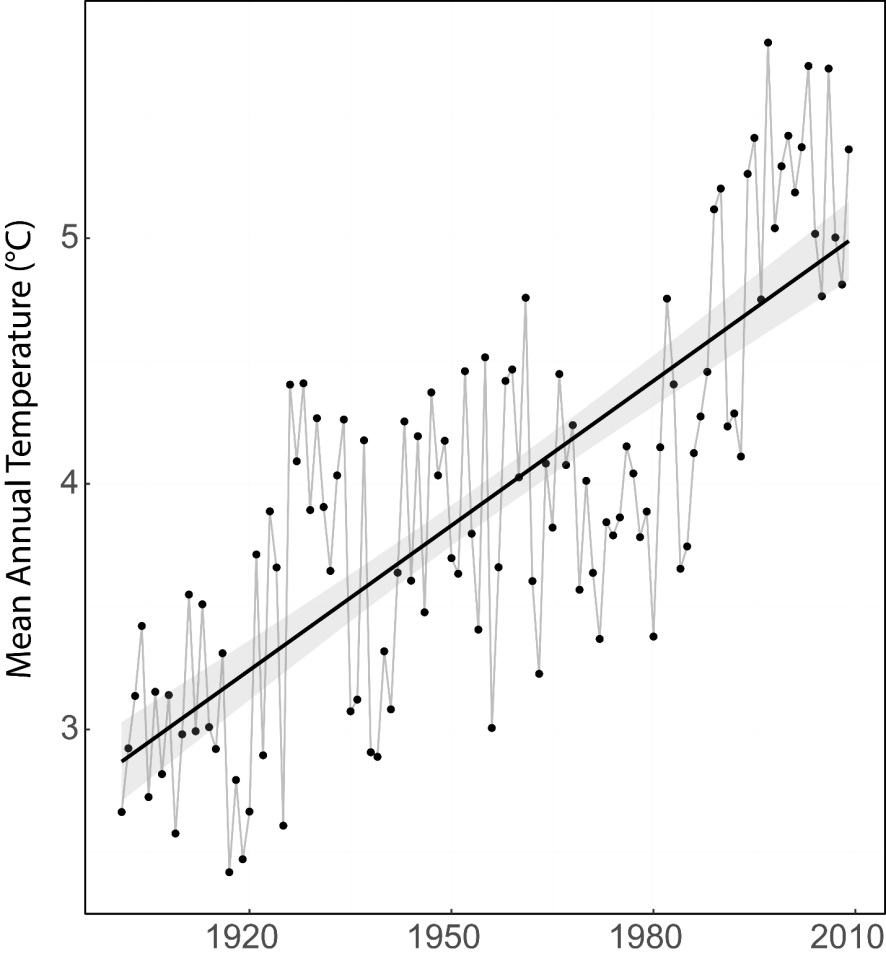


Figure S1 **Mean annual temperature trends between 1900 and 2010.** Line represents linear relationship with 95% confidence interval.


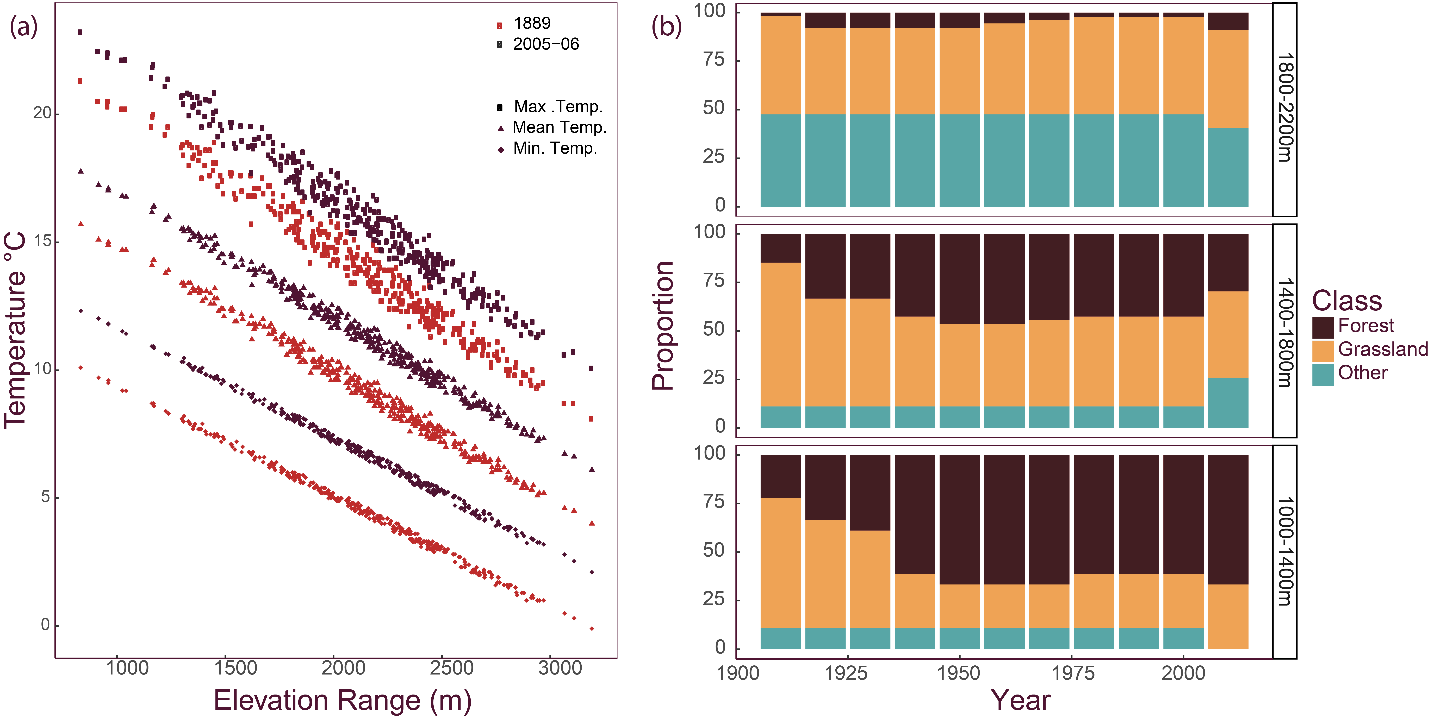


Figure S2 **Abiotic changes between 1900 and 2010 in Luz Valley.** (a) Difference in minimum, mean and maximum temperature changes in August at different elevations between 1900 and 2005-06. (b) Land use change between 1910 and 2010. Reconstructed as part of the Historic Land Dynamics Assessment (HILDA; Fuchs et al. 2013; Fuchs et al. 2015).

**
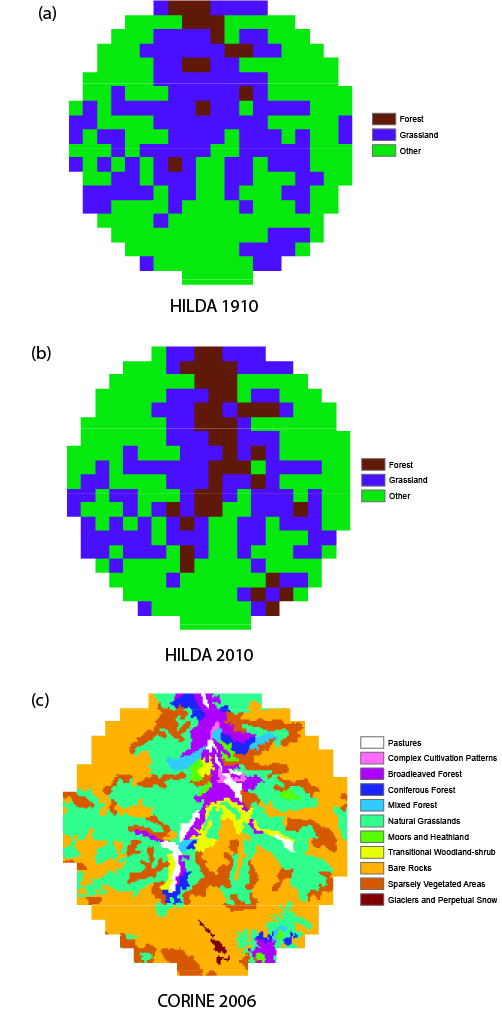
**

Figure S3 **Land use/land cover (LULC) maps Gedre/Gavarnie Pyrenees National Park, 1910, 2010, 2006.** (a) HILDA modelled LULC for 1910; (b) Hilda modelled LULC for 2010; (c) LULC from 2006, CORINE


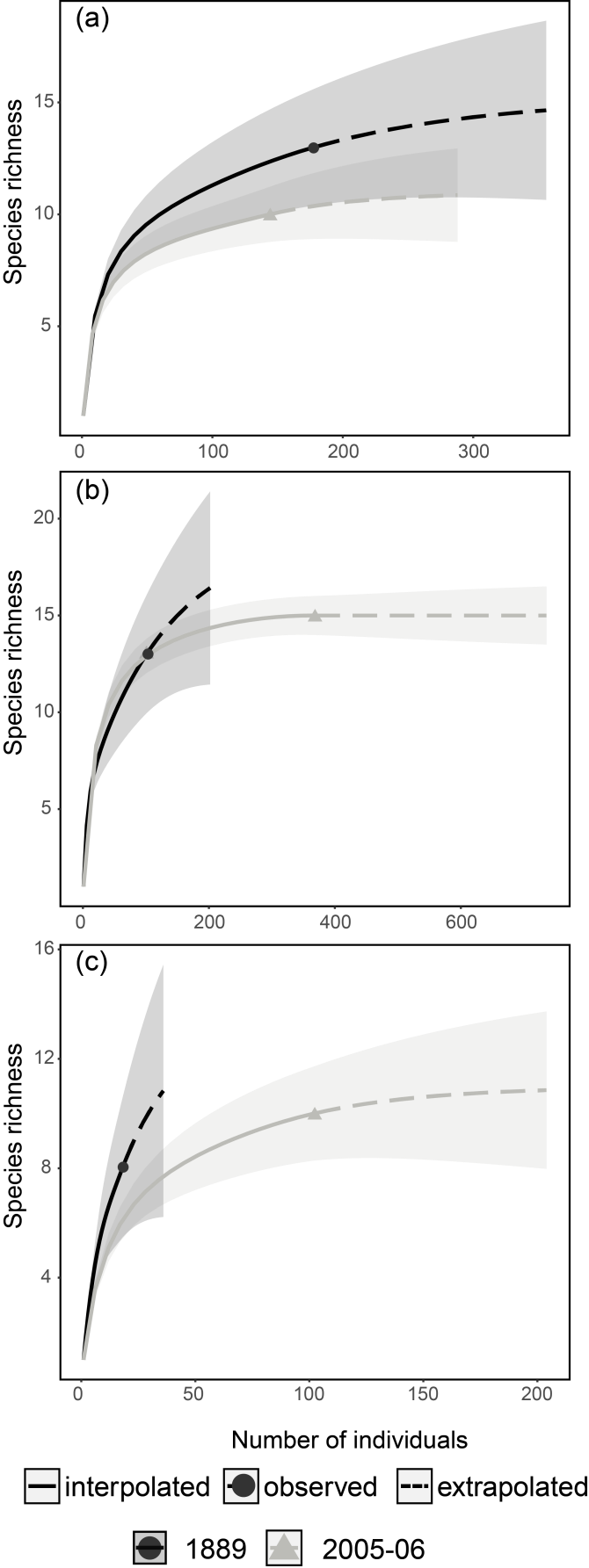


Figure S4 **Bumblebee community diversity and composition in 1889 and 2005-06.** Rarefaction curves of interpolated and extrapolated estimates of species richness (hill number 0) for (a) 1000-1400m, (b) 1400-1800m, and (c) 1800-2200m. Values are extrapolated to twice the number of samples.
